# Neutrophil and Eosinophil Extracellular Traps in Hodgkin Lymphoma

**DOI:** 10.1097/HS9.0000000000000633

**Published:** 2021-09-01

**Authors:** Ivo M. B. Francischetti, Julie C. Alejo, Ranjit Sivanandham, Theresa Davies-Hill, Patricia Fetsch, Ivona Pandrea, Elaine S. Jaffe, Stefania Pittaluga

**Affiliations:** 1 Hematopathology Section, Laboratory of Pathology, Center for Cancer Research, National Cancer Institute, National Institutes of Health (NIH), Bethesda, Maryland, USA; 2 Department of Pathology, School of Medicine, University of Pittsburgh, Pennsylvania, USA; 3 School of Public Health, University of Pittsburgh, Pennsylvania, USA

## Abstract

Classic Hodgkin lymphoma (cHL), nodular sclerosis (NS) subtype, is characterized by the presence of Hodgkin/Reed-Sternberg (HRS) cells in an inflammatory background containing neutrophils and/or eosinophils. Both types of granulocytes release extracellular traps (ETs), web-like DNA structures decorated with histones, enzymes, and coagulation factors that promote inflammation, thrombosis, and tumor growth. We investigated whether ETs from neutrophils (NETs) or eosinophils (EETs) are detected in cHL, and evaluated their association with fibrosis. We also studied expression of protease-activated receptor-2 (PAR-2) and phospho-extracellular signal-related kinase (p-ERK), potential targets/effectors of ETs-associated elastase, in HRS cells. Expression of tissue factor (TF) was evaluated, given the procoagulant properties of ETs. We analyzed 32 HL cases, subclassified as 12 NS, 5 mixed-cellularity, 5 lymphocyte-rich, 1 lymphocyte-depleted, 4 nodular lymphocyte-predominant HL (NLPHL), and 5 reactive nodes. Notably, a majority of NS cHL cases exhibited NET formation by immunohistochemistry for citrullinated histones, with 1 case revealing abundant EETs. All other cHL subtypes as well as NLPHL were negative. Immunofluorescence microscopy confirmed NETs with filamentous/delobulated morphology. Moreover, ETs formation correlates with concurrent fibrosis (*r* = 0.7999; 95% CI, 0.6192-0.9002; *P* ≤ 0.0001). Results also showed that HRS cells in NS cHL expressed PAR-2 with nuclear p-ERK staining, indicating a neoplastic or inflammatory phenotype. Remarkably, TF was consistently detected in the endothelium of NS cHL cases compared with other subtypes, in keeping with a procoagulant status. A picture emerges whereby the release of ETs and resultant immunothrombosis contribute to the inflammatory tumor microenvironment of NS cHL. This is the first description of NETs in cHL.

## Introduction

Classic Hodgkin lymphoma (cHL) is a neoplasm characterized by the presence of clonal malignant Hodgkin/Reed-Sternberg (HRS) cells in a reactive cellular background comprising variable numbers of granulocytes, plasma cells, lymphocytes, and macrophages. In contrast to other hematolymphoid neoplasms in which neoplastic cells represent most of the cellular infiltrate, HRS cells often constitute 0.1%-1% of the cells present.^1–3^ Several contributing factors have been identified in the pathogenesis of cHL, including downregulated expression of B-cell transcription factors in HRS cells, in addition to constitutive activation of NF-kB (nuclear factor kappa-light-chain-enhancer of activated B cells) and Janus kinase (JAK)-signal transducer and activator of transcription (STAT), and amplification of PDL-1 genes. Infectious agents such as Epstein-Barr virus (EBV) also play a major role in cHL pathogenesis, through expression of EBV-encoded latency genes in HRS cells.^1–3^

HRS cells interact and modulate immune cell functions by producing a variety of cytokines, chemokines, and surface ligands that influence specific aspects of the host response; these interactions seem to control both local tumor microenvironment (TME) and systemic manifestations of cHL.^1–3^ On the other hand, the participation of vascular biology has been less often studied in the disease^4–9^ despite its redundant, synergistic, and complementary roles in inflammation and tumor growth.^10,11^ For instance, while neutrophils are commonly found in nodular sclerosis (NS) cHL subtype, the mechanism of accumulation and activation of neutrophils is largely unknown. Answering these questions is relevant given the discovery that granulocytes generate extracellular traps (ETs) designated as neutrophil extracellular traps (NETs) when released by neutrophils, or eosinophil extracellular traps (EETs), when released by eosinophils.^12–14^

NETs are stimulated by numerous factors including bacteria, viruses, or cytokines through a process termed NETosis, that results in extrusion of granulocyte DNA decorated with antimicrobial proteins such as myeloperoxidase (MPO).^12,13^ While these findings were initially described as a novel antimicrobial defense mechanism through killing of invading organisms, numerous reports have provided evidence that NETs promote inflammation as well as tissue injury.^12–14^ For instance, NETs modulate the immune system by triggering autoantibody production, reducing the threshold for T-cell activation^15^ and contributing to loss of immune cells that may be critical to fight infections and cancer.^13^ In addition, NETs promote coagulation through the contact pathway,^16,17^ activate endothelial cells through multiple mechanisms with expression of tissue factor (TF),^13,16^ bind anticoagulant tissue factor pathway inhibitor and have antifibrinolytic activity.^18,19^ NETs are also associated with elastase, a protease that cleaves protease-activated receptor-2 (PAR-2), resulting in MAP kinase pathway activation and phospho-extracellular signal-related kinase (p-ERK).^11,20^ More recently, NETs and NET-associated cell death were implicated in promoting fibrosis.^21^

Given their pleotropic effects, neutrophils, NETs, and inflammation have been pathologically linked to tumor growth.^22,23^ The aims of this study were to determine whether (1) NETs and EETs formation occur in cHL and its association with fibrosis, (2) HRS cells express PAR-2 and show nuclear staining for p-ERK,^20^ and (3) TF, the clotting initiator,^18,19^ is expressed in the neoplastic nodes, given the procoagulant properties of ETs. Our results suggest that inflammatory events collectively described as immunothrombosis take place in NS cHL, and conceivably contribute to the inflammatory TME and fibrotic background seen in the disease.

## Materials and methods

### Case selection

The pathology database of the Laboratory of Pathology, National Cancer Institute, was searched for cases of Hodgkin lymphoma and reactive conditions identified in the archives from 2015 to 2019. This study was approved by the Institutional Review Board of the National Cancer Institute (protocol number NCI-94-C-0074). Thirty-two lymph nodes with adequate tissue were selected for study. The following cHL subtypes were included: 12 NS, 5 mixed-cellularity (MC), 5 lymphocyte-rich (LR), and 1 lymphocyte-depleted (LD) cHL. In addition, 4 nodular lymphocyte-predominant HL (NLPHL) and 5 reactive lymph nodes further designated as follicular hyperplasia (4) and progressive transformation of germinal centers (1) (PTGC) were analyzed. Non-Hodgkin lymphoma cases and other lymphoproliferative disorders were also used for comparison. A summary of the patients’ demographics, site of biopsies, and diagnosis is presented in Table 1. Supplemental Digital Content Table 1S (http://links.lww.com/HS/A191) shows tumor grading (when applicable), HRS cell density/distribution, immunophenotype (CD30, CD15, CD20, PAX5, MUM1), EBER expression, clonality for T-cell receptor and Ig B-cell receptor gene rearrangements, and relevant clinical information.

**Table 1.** Demographics, Biopsy Site, Semi-quantitative Scoring and Necrosis.

Case	Age	Sex	Biopsy Site	MPO	EPX	ETs	PAR-2^*a*^	p-ERK^*a*^	Trich	TF^*b*^	Necrosis
NS cHL											
1	23	M	LN, left supraclavicular	2+/4+	1+/4+	Negative	4+/4+	4+/4+ (v)	1+/4+	2+/4+	Microabcess
2	66	M	LN, subcarinal	3+/4+	1+/4+	2+/4+	4+/4+	4+/4+(s)	3+/4+	1+/4+	Absent
3	31	M	LN, supraclavicular	2+/4+	2+/4+	Negative	4+/4+	4+/4+ (s)	3+/4+	1+/4+	Absent
4	26	M	LN, supraclavicular	4+/4+	2+/4+	1+/4+	4+/4+ (d)	4+/4+ (s)	3+/4+	2+/4+	Absent
5	41	M	LN, left neck	4+/4+	2+/4+	1+/4+	4+/4+	4+/4+ (s)	2+/4+	1+/4+	Incipient
6	63	F	LN, celiac	4+/4+	2+/4+	Negative	4+/4+	2+/4+ (v)	3+/4+	2+/4+	Present
7	74	F	Mediastinal mass	4+/4+	1+/4+	2+/4+	4+/4+	4+/4+ (s)	3+/4+	3+/4+	Present
8	38	M	Mediastinal mass	3+/4+	1+/4+	1+/4+	4+/4+	4+/4+ (s)	3+/4+	1+/4+	Present
9	36	M	LN, left inguinal	4+/4+	1+/4+	1+/4+	1+/4+ (v)	4+/4+(v)	2+/4+	3+/4+	Present
10	67	F	LN, inguinal	3+/4+	2+/4+	1+/4+	0/4+	3+/4+ (v)	2+/4+	3+/4+	Absent
11	63	F	Mass, left neck	4+/4+	4+/4+	2+/4+	4+/4+	4+/4+ (s)	4+/4+	N/A	Present
12	15	F	Anterior mediastinum	4+/4+	2+/4+	2+/4+	N/A	4+/4+ (s)	2+/4+	1+/4+	Absent
MC cHL											
13	28	N/A	LN, cervical	0-1+/4+	0-1+/4+	Negative	4+/4+ (d)	3+/4+(v)	0/4+	Negative	Absent
14	67	F	LN, left neck	0-1+/4+	0-1+/4+	Negative	4+/4+	3+/4+ (v)	0/4+	Negative	Absent
15	47	F	LN, right neck	0-1+/4+	0-1+/4+	Negative	4+/4+	1+/4+ (d)	0/4+	Negative	Absent
16	41	F	LN, hepatic hilar	0-1+/4+	0-1+/4+	Negative	4+/4+	3+/4+ (d)	0/4+	1+/4+	Absent
17	49	M	LN, left cervical	0-1+/4+	0-1+/4+	Negative	4+/4+	4+/4+ (v)	0/4+	Negative	Absent
LR cHL											
18	77	M	LN, right clavicular	0-1+/4+	0-1+/4+	Negative	4+/4+	1+/4+(d)	0/4+	Negative	Absent
19	51	M	LN, cervical	0-1+/4+	0-1+/4+	Negative	4+/4+	0/4+	0/4+	Negative	Absent
20	63	F	LN, left axillary	0-1+/4+	0-1+/4+	Negative	4+/4+ (d)	3+/4+ (v)	0/4+	Negative	Absent
21	42	M	LN, right pelvic	0-1+/4+	0-1+/4+	Negative	4+/4+	0/4+	0/4+	Negative	Absent
22	58	F	LN, supraclavicular	0-1+/4+	0-1+/4+	Negative	4+/4+	3+/4+% (v)	0/4+	Negative	Absent
LD cHL											
23	33	F	LN, posterior cervical	2+/4+	2+/4+	Negative	4+/4+	3+/4+% (v)	0+/4+	Negative	Absent
NLPHL											
24	74	F	LN, left axilla	0-1+/4+	0-1+/4+	Negative	N/A	1+/4+ (d/n)	0/4+	Negative	Absent
25	34	M	LN, left axilla	0-1+/4+	0-1+/4+	Negative	4+/4+	1+/4+ (d/n)	0/4+	N/A	Absent
26	19	M	LN, left inguinal	0-1+/4+	0-1+/4+	Negative	4+/4+	1+/4+(d/n)	0/4+	Negative	Absent
27	24	F	LN, left inguinal	0-1+/4+	0-1+/4+	Negative	4+/4+	3+/4+ (d)	0/4+	Negative	Absent
Reactive lymph nodes											
28	13	M	LN, left inguinal	0-1+/4+	0-1+/4+	Negative	0/4+	0/4+	0/4+	Negative	Absent
29	27	F	LN, left neck	0-1+/4+	0-1+/4+	Negative	0/4+	N/A	0/4+	Negative	Absent
30	55	F	LN, right groin	0-1+/4+	0-1+/4+	Negative	0/4+	N/A	0/4+	Negative	Absent
31	54	M	LN, right jugular level 3	0-1+/4+	0-1+/4+	Negative	0/4+	0/4+	0/4+	Negative	Absent
32	26	F	LN, level 3	0-1+/4+	0-1+/4+	Negative	0/4+	0/4+	0/4+	Negative	Absent

All reactive lymph nodes were cases of follicular hyperplasia, except case #28, diagnosed as PTGC.

^*a*^PAR-2 and p-ERK in HRS cells.

^*b*^TF in endothelial cells.

cHL = classic Hodgkin lymphoma; d = dim; EPX = eosinophil peroxidase; ETs = neutrophil or eosinophil extracellular traps; F = female; HRS = Hodgkin/Reed-Sternberg cells; LD = lymphocyte-depleted; LN = lymph node; LR = lymphocyte-rich; M = male; MC = mixed-cellularity; MPO = myeloperoxidase; N/A = not available; n = negative; NLPHL = nodular lymphocyte-predominant Hodgkin lymphoma; NS = nodular sclerosis; p-ERK = phospho-extracellular signal-regulated kinase; PAR-2 = protease-activated receptor-2; PTGC = progressive transformation of germinal centers; s = strong; TF = tissue factor; Trich. = trichrome; v = variable.

### Automated immunohistochemistry

All formalin-fixed paraffin-embedded (FFPE) tissue biopsies were reviewed by the authors utilizing the criteria of the 2016 World Health Organization (WHO) classification.^1–3^ Immunohistochemistry (IHC) staining for MPO (polyclonal rabbit, DAKO, 1:1000) and eosinophil peroxidase (EPX, 1:1000, a gift from Dr N.A. Lee, Mayo Clinic) were employed to determine the relative abundance of granulocytes. This was done on the Ventana Benchmark Ultra with 64 minute CC1 pretreatment, with 32 minutes incubation with primary antibody, and detection with DAB ultraView. Staining for p-ERK (Phospho-p44/42 mitogen-activated protein kinase [MAPK] [Erk1/2] [D13.14.4E] XP Rabbit mAb #4370, Cell Signaling) at 1:500, and TF (rabbit polyclonal anti-TF, PA5-27278, Thermo Fisher) at 1:400 or 0.4 µg/mL was performed on FFPE on Leica BOND-MAX (Leica Microsystems, Bannockburn, IL) automated immunostainer. Antigen retrieval was performed with BOND epitope Retrieval Solution 2 (EDTA-based pH 9.0, for heat-induced retrieval) or standard DAB protocol.^24,25^ Fibrosis was detected with Masson’s trichrome stain. Semiquantitative scoring for TF, p-ERK, and fibrosis was as follows: rare or absent (0, or negative), <10% (1+), 11%-30% (2+), 31%-50% (3+), >50% (4+). Images were obtained with Nikon DS-Fi-3 camera coupled to an Olympus BX50 microscope. Image files were collected with Software NIS-Elements D, version 5.11.

### IHC for NETs and PAR-2

For detection of NETs, we used an antibody for citrullinatedhistones. Citrullination, also termed deimination, is a post-translational protein modification catalyzed by the Ca^2+^-dependent peptidyl arginine deiminases, an enzyme necessary for formation of NETs.^12,13^ Immunohistochemical staining was performed on FFPE tissue slides. Slides were deparaffinized in xylene (3 × 5 minutes) and rehydrated in graded alcohol (2 × 2 minutes in 95% EtOH [ethanol], 1 × 2 min in 80% EtOH) and distilled water (2 × 2 min). For NETs staining, heat-induced antigen retrieval was performed using Dako Target Retrieval Solution, Citrate pH 6.1 (S1699). Solution was preheated in a pressure cooker (Tender Cooker 62104, Nordic Ware, MN) and placed in microwave oven until hissing (~100°C). Then, the slides were placed in the pressure cooker and microwaved, timing for 6 minutes once hissing began. Subsequently, slides were rinsed in Tris buffer solution and immersed in 3% Tris-goat solution to block nonspecific binding. The primary antibody, anti-citrullinated histone (Abcam, ab5103, a rabbit polyclonal to Histone H3 - citrulline R2 + R8 + R17, 1 mg/mL stock solution) was applied at 1:200 dilution (or 5 µg/mL), and incubated overnight at 4°C. Slides were washed and incubated with anti-rabbit (DAKO K4003) secondary antibody for 30 minutes, at room temperature. Dako Liquid DAB+ Substrate Chromogen System Code (K3468) was used as the detection chromogen and the slides were counterstained with hematoxylin. Semiquantitative scoring for NETs reflected the proportion of positive cells/total granulocytes as follows: rare or absent (0, or negative), <10% (1+), 11%-30% (2+), 31%-50% (3+), >50% (4+).

For PAR-2 staining, slides were placed in Dako Target Retrieval Solution, Citrate pH 6.1 (S1699) and steamed for 30 minutes (~100°C), followed by rinsing in Tris buffer solution. The slides were immersed in 3% Tris-goat solution to block nonspecific binding. The primary antibody, anti-PAR-2 (Abcam, ab184673, mouse monoclonal to PAR-2 clone SAM11, 1 mg/mL stock solution), was applied at 1:400 dilution (or 2.5 µg/ml) and incubated overnight at 4°C. Slides were washed in Tris buffer, and followed by 30 minutes incubation with anti-mouse (DAKO K4001) secondary antibody. Dako Liquid DAB+ Substrate Chromogen System Code (K3468) was used as the detection chromogen and the slides were counterstained with hematoxylin. Scoring for PAR-2 was similar as above. Table 2 summarizes specifics for immunostains, including type of antibody, dilution, source, catalog number, and antigen retrieval details.

**Table 2.** Antibodies and Conditions for Immunohistochemistry.

Antigen	Antibody	Dilution	Incubation	Source	Catalog Number	Retrieval Conditions
Citrullinated histones	Rabbit polyclonal	1 to 200	4°C overnight	Abcam	ab5103	Citrate pH 6.1, heat-induced
Eosinophil peroxidase	Mouse monoclonal (MM25-82.2.1)	1 to 1000	32 min, RT	Dr Lee (Mayo Clinic, AZ)	NA	Leica Protocol
Myeloperoxidase	Rabbit polyclonal	1 to 1000	32 min, RT	Dako	A0398	Roche Protocol
PAR-2	Mouse monoclonal (clone SAM11)	1 to 400	4°C overnight	Abcam	ab184673	Citrate pH 6.1, heat-induced
p-ERK	Rabbit monoclonal (clone D13.14.4E)	1 to 500	32 min, RT	Cell Signaling	mAb#4370	EDTA-based pH 9.0, heat-induced
Tissue factor	Rabbit polyclonal	1 to 400	32 min, RT	ThermoFisher	PA5-27278	EDTA-based pH 9.0, heat-induced

NA = not applicable; p-ERK = phospho-extracellular signal-regulated kinase; PAR-2 = protease-activated receptor-2; RT = room temperature.

### Immunofluorescence for NETs

Immunofluorescence (IF) staining for NETs was performed on FFPE tissue samples as previously described.^13^ Four µm thick sections were deparaffinized and rehydrated. For MPO staining, antigen retrieval was performed at pH 6.0 in Vector Unmasking Solution (Vector Laboratories, Burlingame, CA) and boiled in a microwave oven for 20 minutes. Sections were incubated with the anti-MPO primary antibody (1:800 dilution) (A0398, Agilent Technologies) followed by incubation with donkey anti-rabbit conjugated with Alexa Fluor 488 (Abcam) (1:100 dilution). For neutrophil elastase (NE), antigen retrieval was done by incubating the tissues at pH 6.0 in Vector Unmasking Solution (Vector Laboratories) at 50°C for 90 minutes, followed by an incubation with anti-NE primary antibody (1:50 dilution) (Agilent Technologies) and then with a goat anti-mouse secondary antibody conjugated with Alexa Fluor 568 (1:150 dilution). The tissues were then incubated with DAPI (4′,6-diamidino-2-phenylindole) (1:5000 dilution) (Millipore Sigma, Burlington, MA) for nuclear staining, followed by Trueblack lipofuscin autofluorescence quencher (1:20 dilution in 70% ethanol) (Biotium Inc, Hayward, CA), to suppress autofluorescence. Slides were cover-slipped using fluorescent mounting media (Agilent) and coverslips (Thermo Fisher Scientific) and imaged on an Olympus Fluoview 1000 Confocal Microscope. Images were processed with NIS-Elements AR 5.2 (Nikon).

### Statistical analysis

Descriptive statistics are expressed as mean ± standard error of the mean. For variables with nonparametric distributions, the Kruskal-Wallis test (followed by Dunn pairwise post hoc comparisons) was employed. Spearman rank coefficient correlation was used to test the association between quantitative variables. A *P* value of ≤0.05 was considered significant (GraphPad Prism version 8.4.2).

## Results

**Figure 1.**
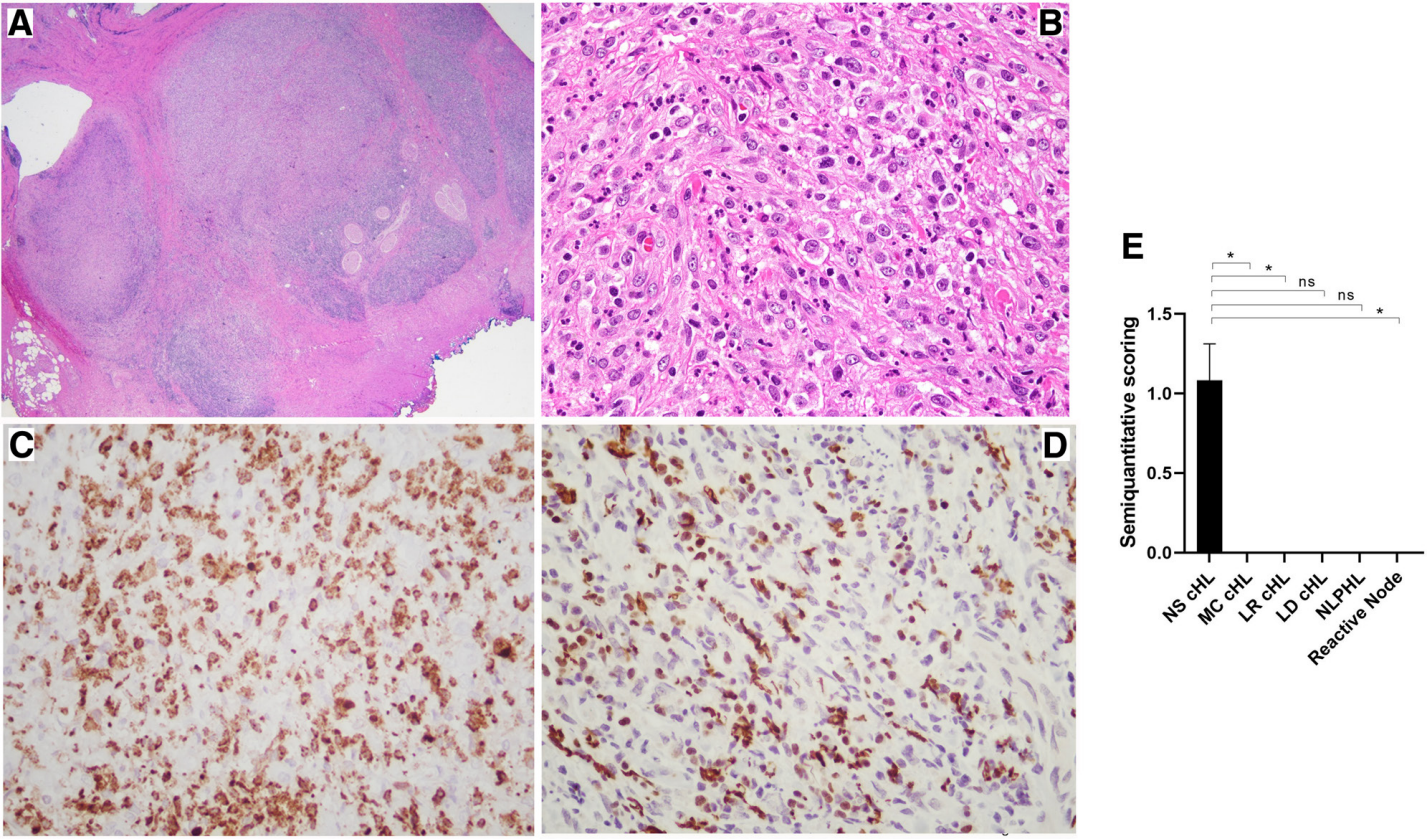
**NETs in NS cHL.** NS cHL (case #2). (A) H&E shows an atypical lymphoid proliferation with a nodular growth pattern, surrounded by dense fibrous bands (×20). (B) Scattered HRS-like cells, numerous neutrophils, and scattered eosinophils (×200). (C) MPO staining shows areas of neutrophilic infiltration (×200). (D) Polyclonal antibody for citrullinated histone shows high power view of NETs with different morphologies including neutrophils in the initial stages of NETosis, and neutrophils that had already ejected their condensated chromatin into the extracellular space (×200). (E) Semiquantitation of ETs formation in NS cHL and other conditions. Results are expressed as mean ± SEM (*P ≤ 0.05). cHL = classic Hodgkin lymphoma; ET = neutrophil or eosinophils extracellular traps; H&E = hematoxilin & eosin; HRS = Hodgkin/Reed-Sternberg cells; LD = lymphocyte-depleted; LR = lymphocyte-rich; MC = mixed-cellularity; MPO = myeloperoxidase; NETs = neutrophil extracellular traps; ns = nonsignificant; NS = nodular sclerosis; SEM = standard error of the mean.

Using IHC with antibodies for citrullinated histones, we show NETs or EETs in most cases of NS cHL, and confirm their association with neutrophilic or eosinophilic infiltration. Accordingly, in 9 of 12 NS cHL cases studied (75%) NETs were present in a subset of neutrophils, with variable positivity and focal distribution. In contrast, NETs were not detected in MC cHL (0/5), LR cHL (0/5), LD cHL (0/1), NLPHL (0/4), or in any reactive conditions tested (0/5) (Table 1), indicating that this process is commonly seen in NS cHL. Figures 1A, B illustrate a typical case of NS cHL. Infiltrating neutrophils are highlighted with staining for MPO (Figure 1C), and abundant NET formation with the typical lobulated, delobulated, and filamentous morphology is illustrated in Figure 1D.^20^ Detection of NETs was statistically significant for NS cHL versus MC cHL (*P* = 0.035), LR cHL (*P* = 0.035), reactive conditions (*P* = 0.035), and nonsignificant for NLPHL (*P* = 0.07) and LD cHL (*P* > 0.99) (Figure 1E).

**Figure 2.**
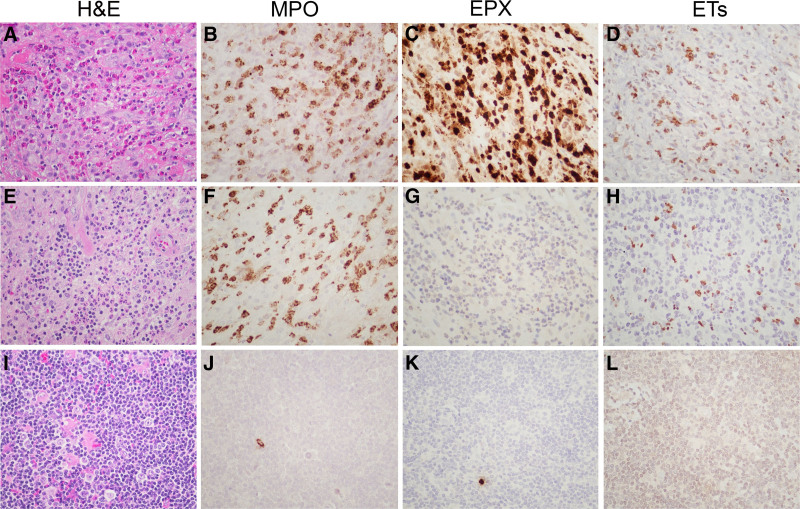
**EETs in NS cHL.** NS cHL (case #11) (A–D). (A) Tissue eosinophilia and rare neutrophils. (B) MPO staining marks areas of granulocytic infiltration. (C) EPX staining reveals massive eosinophilia. (D) EETs staining associated with eosinophilic infiltration. H&E of NS cHL (case #8) (E–H). (E) Marked tissue neutrophilia and few eosinophils. (F) MPO staining marks granulocytic infiltration. (G) EPX staining reveals rare eosinophils. (H) NETs staining associated with neutrophilic infiltration. NLPHL (case #25) (I–L). (I) Pauciinflammatory background and rare neutrophils or eosinophils. (J) MPO staining shows rare granulocytes. (K) EPX staining marks rare eosinophils. (L) NETs staining is undetectable in the absence of granulocytes. All images (×200). cHL = classic Hodgkin lymphoma; EET = eosinophil extracellular traps; EPX = eosinophil peroxidase; ET = neutrophil or eosinophils extracellular traps; H&E = hematoxilin & eosin; MPO = myeloperoxidase; NETs = neutrophil extracellular traps; NLPHL = nodular lymphocyte-predominant Hodgkin lymphoma; NS = nodular sclerosis.

To determine whether tissue eosinophilia was associated with EETs, 1 case was selected based on an abundance of eosinophils and rare neutrophils on H&E (Figure 2A). This cellular composition was confirmed by similar staining with MPO (Figure 2B) and EPX (Figure 2C). Notably, IHC revealed positive staining in areas infiltrated by >95% eosinophils, indicative of ET formation from eosinophilic derivation (Figure 2D). For comparison, NS cHL case (Figure 2E) with abundance of neutrophils (MPO, Figure 2F) and rare eosinophils (EPX, Figure 2G) demonstrated NET formation of neutrophil derivation (Figure 2H). In contrast, NLPHL (Figure 2I) shows only rare neutrophils (Figure 2J) and eosinophils (Figure 2K), and undetectable NETs (Figure 2L). Additional negative cases comprising PTGC (Supplemental Digital Content, Figure S1A-H, http://links.lww.com/HS/A190) and mixed cellularity cHL (Supplemental Digital Content, Figure S1I-K, http://links.lww.com/HS/A190) are illustrated. Note that mixed cellular cHL contains prominent clusters of neutrophils, but staining for NETs is negative.

**Figure 3.**
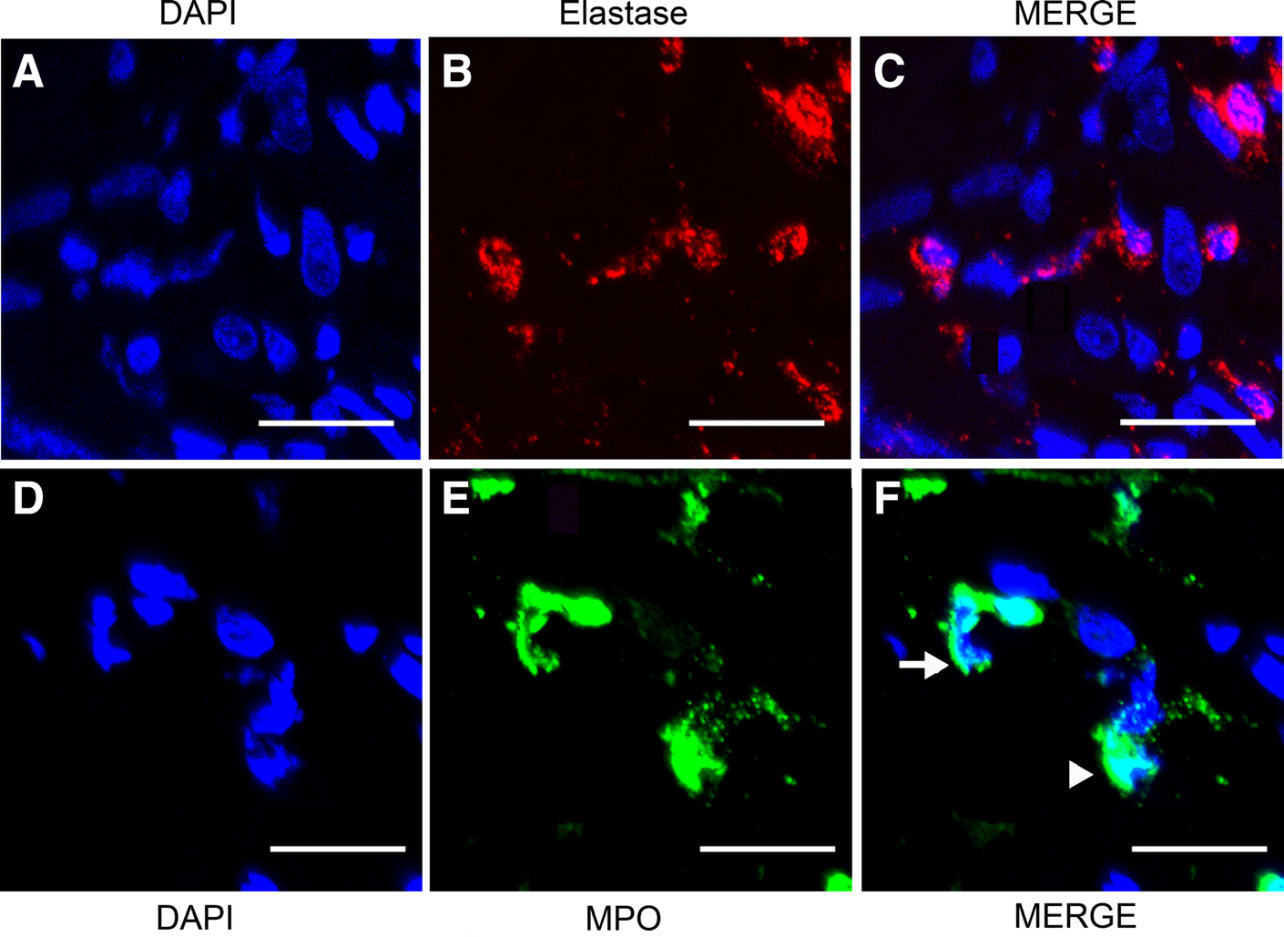
**NETs in NS cHL, by immunofluorescence microscopy.** NS cHL (case #2). (A) Nuclear material identification by DAPI (blue). (B) Visualization of neutrophil elastase (red). (C) Merge of DAPI-stained DNA and elastase shows colocalization. (D) Nuclear material identification by DAPI (blue). (E) Visualization of neutrophil MPO (green). (F) Merge of DAPI-stained DNA and MPO shows colocalization. The arrow shows decondensated chromatin and the arrowhead shows condensated (lobulated) chromatin, respectively. Scale bars are 20 µm in length. cHL = classic Hodgkin lymphoma; DAPI = 4′,6-diamidino-2-phenylindole; NETs = neutrophil extracellular traps; MPO = myeloperoxidase; NS = nodular sclerosis.

NETosis was further confirmed in a subset of 8 cases by IF using anti-elastase or anti-MPO antibodies colocalizing with nuclear material stained by DAPI (Figures 3A, D), a gold-standard for NETs visualization.^13^ This analysis allowed us to identify (Figure 3, NS cHL #2) the characteristic NET filaments/delobulation,^20^ in which neutrophil-derived proteins such as elastase (Figure 3B) and MPO (Figure 3E) colocalized with extracellular DNA labeled with DAPI (Figure 3C, F). Similar results were observed for NS cHL cases #4, #7, #8 (not shown), while case #6 was negative (also by IHC). In contrast, NLPHL case #25 did not show elastase or MPO staining associated with extracellular DNA (Figure S2), a negative result also noted for reactive conditions cases #28 and #32 (not shown).

**Figure 4.**
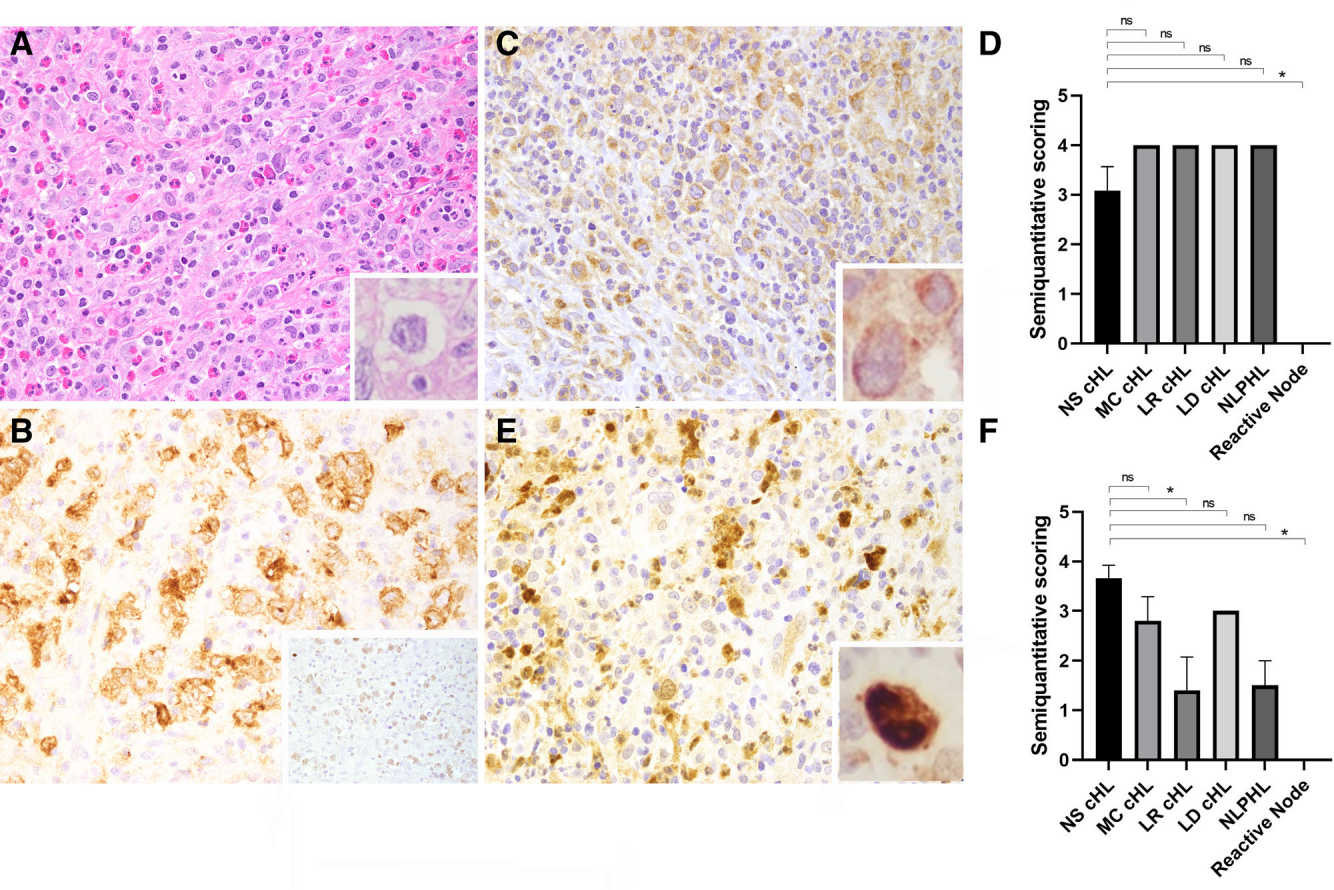
**PAR-2 and p-ERK expression in NS cHL.** NS cHL (case #8). (A) H&E shows HRS cells and interspersed eosinophils and background fibrosis (inset, ×1000). (B) HRS cells shows membranous staining for CD30 and dim nuclear PAX-5 (inset, ×200). (C) HRS cells shows perinuclear expression of PAR-2 (inset, ×1000). (D) Semiquantitation of PAR-2 expression in NS cHL and other conditions. (E) HRS cells show nuclear p-ERK staining (inset, ×1000). (F) Semiquantitation of p-ERK expression in NS cHL and other conditions. Results are expressed as mean ± SEM (*P ≤ 0.05). cHL = classic Hodgkin lymphoma; H&E = hematoxilin & eosin; HRS = Hodgkin/Reed-Sternberg cells; LD = lymphocyte-depleted; LR = lymphocyte-rich; MC = mixed-cellularity; ns, nonsignificant; NS = nodular sclerosis; p-ERK = phospho-extracellular signal-regulated kinase; PAR-2 = protease-activated receptor-2; SEM = standard error of the mean.

PAR-2 is a proinflammatory receptor and potential target for NETs-associated elastase.^11^ Overall, ≥50% of HRS cells expressed PAR-2 as follows: NS cHL (11/12, 91%), MC cHL (5/5, 100%), LR cHL (5/5, 100%), LD cHL (1/1, 100%), and LP cells of NLPHL (3/3, 100%)(Table 1). Figures 4A, B show a typical cHL case. Figure 4C shows that PAR-2 staining in HRS cells display a perinuclear/cytoplasmic pattern. PAR-2 was also positive in LR cHL and other subtypes of cHL although less intense in LP cells of NLPHL (not shown). Additionally, PAR-2 was positive in a subset of mid-sized lymphoid cells, indicating that it is not specific for HRS cells, thus limiting its use for diagnostic purposes. No statistically significant difference in PAR-2 staining was observed for NS cHL versus other subtypes and NLPHL (*P* > 0.99), except for reactive conditions (*P* = 0.003) (Figure 4D).

ERK is part of the MAPK (mitogen-activated protein kinase) pathway that mediates cytokine production, cell proliferation, and differentiation.^11^ Overall, more than 50% of HRS cells expressed mostly strong nuclear p-ERK in NS cHL (12/12, 100%), as depicted for 1 case in Figure 4E. p-ERK positivity was also seen in most cHL subtypes, although with somewhat variable staining intensity (Table 1). LP cells of NLPHL were essentially negative (or dim) for p-ERK, as well as reactive conditions (not shown). No statistically significant difference in p-ERK staining was observed for NS cHL versus MC cHL, LD cHL (*P* > 0.99) and NLPHL (*P* = 0.10), but it was for LR cHL (*P* = 0.027) and reactive conditions (*P* = 0.0036) (Figure 4F).

**Figure 5.**
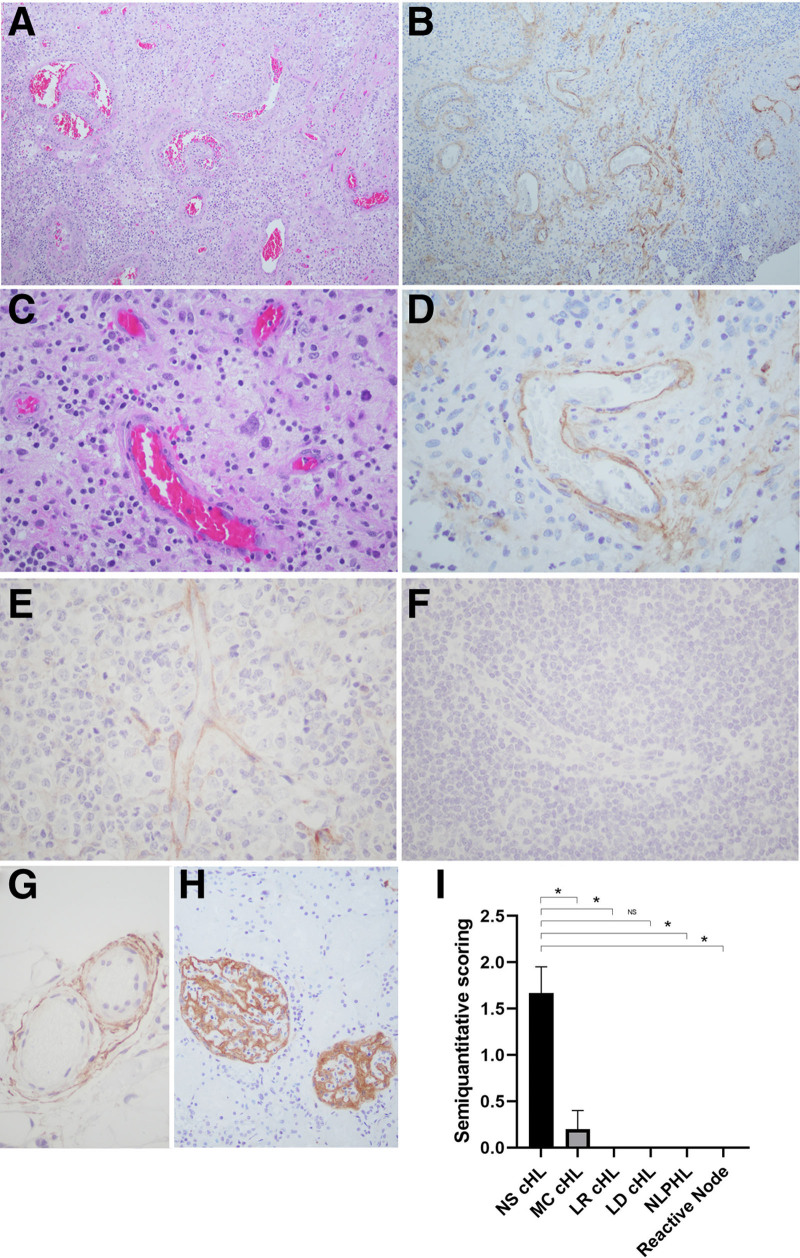
**TF staining in the endothelium of NS cHL.** NS cHL (case #7) (A–D). (A) H&E of lymph node with numerous HRS cells (high-density tumor areas) (×100) and vessels. (B) Positive staining for TF in the endothelial cells in high-density tumor areas (×100). (C) H&E of lymph node with numerous HRS cells (high-density tumor areas) and vessels (×400). (D) Positive staining for TF in the endothelial cells in high-density tumor areas (×400). (E) NS cHL (case #11). Positive staining for TF in endothelium (×400). (F) LR cHL (case #19). Negative staining for TF in endothelium (×400). (G) Control TF staining in the perinodal fat tissue is consistently detected in the adventitia around small- and medium-sized vessels, but not in the endothelium (×400). (H) Positive staining for TF in the glomeruli of an autopsy case (×200). (I) Semiquantitation of TF staining in NS cHL and other subtypes and reactive conditions. Results are expressed as mean ± SEM (*P ≤ 0.05). No staining was observed in the absence of primary antibody (not shown). cHL = classic Hodgkin lymphoma; H&E = hematoxilin & eosin; HRS = Hodgkin/Reed-Sternberg cells; LD = lymphocyte-depleted; LR = lymphocyte-rich; MC = mixed-cellularity; NLPHL = nodular lymphocyte-predominant Hodgkin lymphoma; ns = nonsignificant; NS = nodular sclerosis; SEM = standard error of the mean; TF = tissue factor.

NETs affect the coagulation cascade through multiple mechanisms.^14,16^ For instance, DNA activates the intrinsic (contact) pathway leading to thrombin production. Other components such as histones and elastase induce TF expression in endothelial cells resulting in coagulation activation by the extrinsic pathway. To verify TF staining, all specimens in our study were probed with an anti-TF antibody. We identified positive endothelial TF staining in 100% of NS cHL cases available (11/11). In contrast, staining was predominantly negative in 19 of 20 other cases (95%), including MC cHL (1/5, 20%), LR cHL (0/5, 0%), LD cHL (0/1, 0%), NLPHL (0/4, 0%), and reactive conditions (0/5, 0%) (Table 1). Figure 5A–F shows representative results and highlight staining of endothelium TF in NS cHL HRS cell-rich areas, but not in HRS cell-poor areas, or other subtypes. Numerous controls validated our study. For instance, normal staining for TF was present in the adventitia around small to medium sized vessels (Figure 5G) and in the kidney glomeruli (Figure 5H).^26,27^ Detection of TF was statistically significant for NS cHL versus MC cHL (*P* = 0.031), LR cHL (*P* = 0.003), NLPHL (*P* = 0.037), reactive conditions (*P* = 0.003), and nonsignificant for LD cHL (*P* = 0.88) (Figure 5I).

Our findings for TF staining were remarkably consistent among NS cHL cases as shown in the Supplemental Digital Content (http://links.lww.com/HS/A190). Supplemental Digital Content Figure S3A reveals HRS cell-rich areas associated with endothelial staining for TF (Supplemental Digital Content, Figure S3B). In the same slide, areas of the node containing few or no HRS cells (Supplemental Digital Content, Figure S3C) were negative for TF staining. To determine whether TF staining is commonly found in other lymphoproliferative disorders, 10 more lymph nodes were evaluated with anti-TF antibody. These other conditions include Kikuchi-Fujimoto lymphadenopathy, reactive follicular- and paracortical hyperplasia, NLPHL with features of T-cell/histiocyte-rich B-cell lymphoma, EBV-positive lymphoproliferative disorders, chronic active EBV-infection of B-cell and T-cell types and systemic EBV-positive T-cell lymphoma of childhood. In all cases, endothelial staining for TF was consistently negative (not shown).

**Figure 6.**
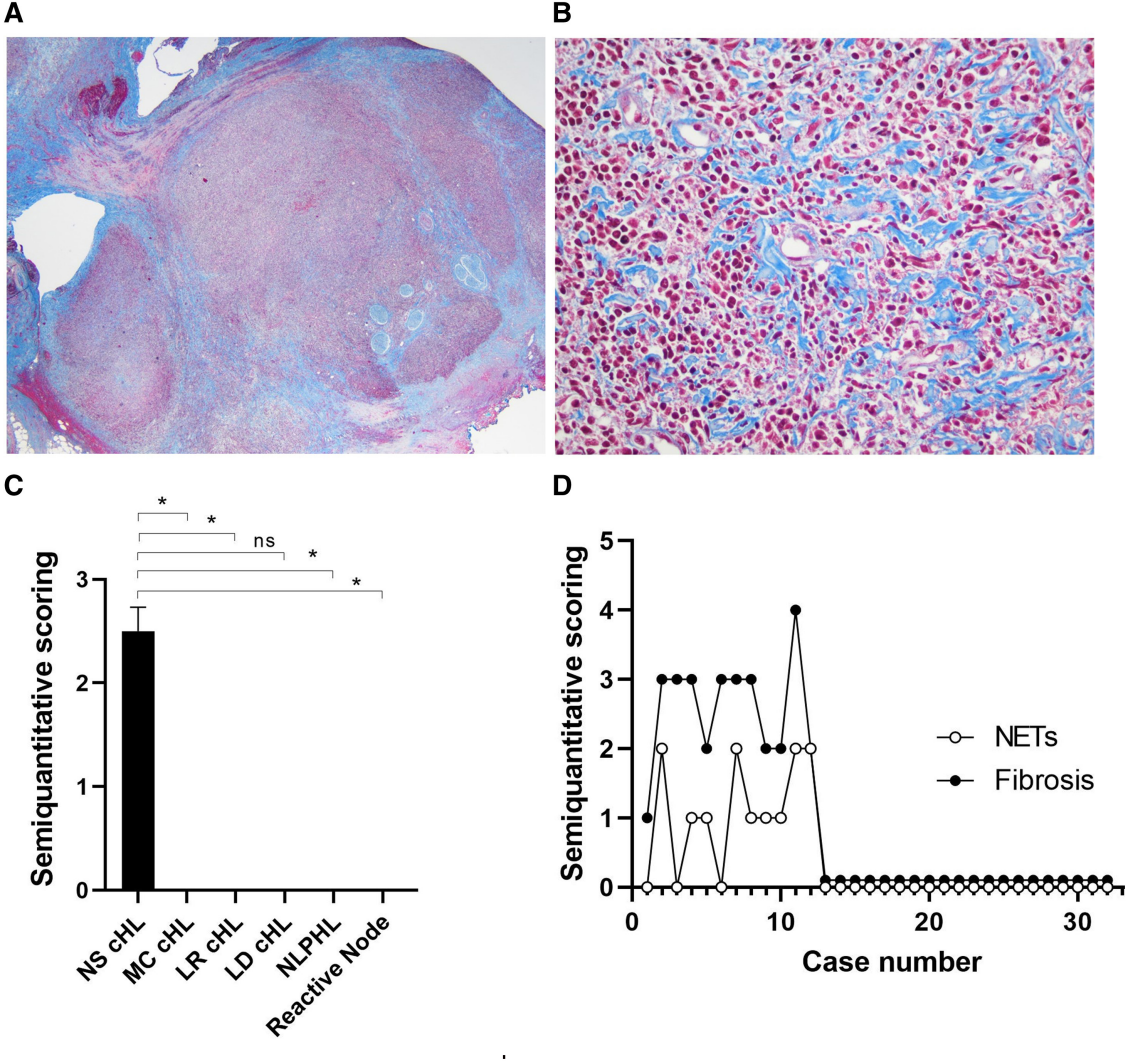
**Fibrosis in NS cHL, and association with NETs.** NS cHL (case #2). (A) Masson Trichrome’s staining at low power shows a nodular configuration (×20) and (B) depicts a interstitial pattern (×200). (C), Semiquantitation of fibrosis in NS cHL and other conditions. Results expressed as mean ± SEM (*P ≤ 0.05). (D) Spearman’s correlation of NETs and fibrosis (*r* = 0.7999; 95% CI, 0.6192-0.9002; *P ≤* 0.0001). cHL = classic Hodgkin lymphoma; LD = lymphocyte-depleted; LR = lymphocyte-rich; MC = mixed-cellularity; NETs = neutrophil extracellular traps; NLPHL = nodular lymphocyte-predominant Hodgkin lymphoma; ns = nonsignificant; NS = nodular sclerosis; SEM = standard error of the mean.

Fibrosis is often associated with cHL, NS subtype, and the extent of node involvement is likely secondary to the time of disease involvement and progression. All specimens were stained with Masson’s-trichrome given the association of fibrosis with NET formation.^21^ We found that every NS cHL case positive for NETs exhibited intense fibrosis (≥2+/4+) (9/9, 100%). In contrast, all other subtypes of cHL (0/11, 0%), NLPHL (0/4, 0%), and reactive conditions (0/5, 0%) were negative for both stains (Table 1). Figure 6A shows a typical case of NS cHL with nodular fibrosis, and Figure 6B depicts the interstitial distribution. Presence of fibrosis was statistically significant for NS cHL versus MC cHL (*P* = 0.003), LR cHL (*P* = 0.003), NLPHL (*P* = 0.009), reactive conditions (*P* = 0.003) but nonsignificant for LD cHL (*P* = 0.87) (Figure 6C). Figure 6D reveals that NET formation and fibrosis have a positive correlation (Spearman *r* 0.7999; 95% confidence interval [CI], 0.6192-0.9002; *P* value ≤0.0001). Finally, slides were scored for the presence of necrosis on H&E. Among 9 NS cHL cases positive for NETs, 5 presented with necrosis, including 1 of incipient type. Among 3 NETs negative cases, 1 had necrosis.

**Figure 7.**
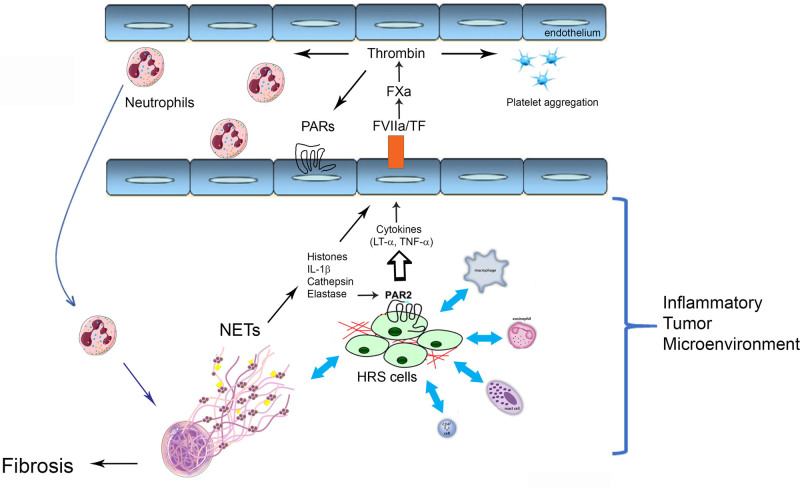
**Immunothrombosis and inflammatory TME in NS cHL.** HRS cells interaction with numerous inflammatory cells support the development of the TME.^1–3^ NETs contribute to tumor growth, through different mechanisms^12–14^ (see Discussion). cHL = classic Hodgkin lymphoma; HRS = Hodgkin/Reed-Sternberg cells; IL = interleukin-1beta; LT = lymphotoxin-alpha; NETs = neutrophil extracellular traps; NS = nodular sclerosis; PAR = protease-activated receptor; TF = tissue factor; TME = tumor microenvironment; TNF = tumor necrosis factor-alpha.

Figure 7 shows a working hypothesis for the potential role of NETs, coagulation, cytokines, proteases, TF, and immunothrombosis^28^ in the development of NS cHL inflammatory TME (see Discussion).

## Discussion

NS cHL is characterized by an inflammatory background and marked fibrosis with well-developed fibrous bands. The pathogenic mechanisms leading to this classic histology and their significance in pathogenesis are not well known. We show that NS cHL (i) is commonly associated with neutrophilic inflammation and NET formation, while other subtypes are negative for NETosis, (ii) a subset with predominance of eosinophils shows EETosis, (iii) NET formation is commonly associated with fibrosis, (iv) HRS cells express cytoplasmic PAR-2 and nuclear p-ERK staining, and (v) TF staining of endothelium is usually present and associated with HRS cell-rich areas. Given the pleiotropic effects of NETs, these findings suggest that it contributes to the inflammatory TME in NS cHL. Identification of NETs in NS cHL indicates that signals required for NETosis are present in the neoplastic tissue. Accordingly, stimulation of neutrophils through toll-like receptor (TLRs), Fc-, cytokine- or complement-receptors in addition to cytokines (eg, IL-8, TNF, and IFN-γ [interleukin-8, tumor necrosis factor, interferon-γ]) can induce NETosis. Most of these molecules have been reported in cHL,^1–3^ and conceivably synergize to promote release of NETs, through multiple mechanisms. For instance, in autoimmune diseases (eg, lupus, rheumatoid arthritis, and psoriasis), NETs stimulate TLR-9 in plasmacytoid dendritic cells to produce IFN-α, among other cytokines.^29^ However, the role of NETs in lymphomagenesis remains incompletely understood. For instance, it has been shown that defective stromal remodeling and NETs in lymphoid tissues favor the transition from autoimmunity to lymphoma.^30^ Another study determined that stromal cell-derived IL-8 induces NET formation and cooperatively sustains follicular lymphoma B-cell growth.^31^ In addition, a recent paper reported that diffuse large B-cell lymphoma cells produce IL-8 to stimulate NET formation, which leads to TLR-9 activation in neoplastic cells. This results in tumor progression and was associated with poorer prognosis in a cohort of 233 patients.^32^ In myeloproliferative disorders with JAK2^V617F^ mutation, NET formation is associated with thrombosis that may occur in these patients.^33^ Altogether, these findings indicate that NETs are linked with pathological responses in distinct malignant hematolymphoid neoplasms, and are not unique to NS cHL.

With respect to NS cHL, we have unequivocally demonstrated NET formation by 2 unrelated techniques. By IHC, NETs were detected in 75% of NS cHL cases with a typical staining pattern characterized by filamentous morphology.^34^ NET formation was often focal and detectable only in a subset of neutrophils, indicating that it is not an equivalent of neutrophilic inflammation; this selectivity might be a result of neutrophil heterogeneity, age, density and/or tightly regulated NETosis.^35^ In contrast, MC cHL, LR cHL, LD cHL, NLPHL, and reactive conditions were consistently negative for NETs. Our results also revealed EETs in NS cHL case #11, which was remarkable for massive eosinophilic infiltration (>95% overall). These findings indicate that both types of granulocytes are sources of ETs in the disease. It is concluded that NET formation is commonly seen in NS cHL, but not in other histological subtypes. It remains to be determined how the presence of ETs correlate with immunophenotype, clinical presentation, and response to treatment (Supplemental Digital Content, Table 1S, http://links.lww.com/HS/A191).

IF microscopy showed elastase physically associated with NETs in NS cHL as illustrated in case #2, with a typical filamentous or delobulated morphology. IF findings were similar to IHC results for NETs detected with anti-citrullinated histones in 7 other cases: positive in NS HL cases #4, #7, #8; negative for NS HL case #6, NLPHL case #25, and for reactive condition cases #28 and #32. The identification of elastase in NS cHL is particularly significant given its proinflammatory and pleiotropic effects in tumor growth.^22^ Accordingly, elastase remodels the matrix and activates dormant tumor cells,^36^ or engages the TLR4-p38-PGC-1α axis resulting in increased proliferation and survival of cancer cells.^37^ Furthermore, elastase proteolytically processes IL-1 family members into active forms and acts as a PAR-2 agonist, with generation of IL-6, IL-8, and IL-1β with upregulation of adhesion/integrin receptors. Notably, HRS cells consistently express PAR-2 at protein and transcriptional levels, suggesting this receptor is a potential target for NET-associated elastase, among other proteases (eg, FXa).^5^ While our report is the first to show elastase in cHL, evidence for elastase activity has been provided for both B-cell non-Hodgkin lymphomas, and T-cell lymphomas,^38,39^ corroborating the view that this enzyme is biologically active in hematopoietic tumors. Coagulation proteases may also contribute to the TME. For instance, NET-associated TF may initiate the clotting cascade on binding to FVIIa, which may also activate PAR-2^13,14,16^. Of note, clotting factors are detectable in the lymph, indicating that they permeate lymph nodes.^40^ Additionally, thrombin generated in the lymphatic compartment has been shown to affect nodal dendritic cell function through PAR-1 in a murine model of severe sepsis.^41^ It is conceivable that the PARs/proteases axis modulates HRS cell function, contributing to the TME as described for other malignancies.^11^ Mechanistic studies are required, however, to determine the relative contribution of proteases in cHL lymphomagenesis.

PAR activation by elastase and FXa is associated with signaling through the MAPK cascade and ERK phosphorylation.^20^ Our results demonstrated p-ERK in the nuclei of HRS cells from NS cHL, consistent with a proinflammatory/neoplastic phenotype. We extended these observations by revealing that HRS cells in MC cHL and LD cHL also exhibit nuclear p-ERK staining. However, staining in these subtypes was generally variable/dimmer and took place in a relatively fewer proportion of HRS cells, in a background with less inflammation, compared with NS cHL. Nevertheless, our results are not conclusive regarding an association between NETs and p-ERK expression, and further studies are required to address this question. Interestingly, p-ERK was mostly dim or negative in HRS cells of LR cHL, and in LP cells of NLPHL. These results are in line with immunophenotypic similarity observed between LR cHL and NLPHL, and a pauciinflammatory microenvironment in both entities.^1–3^

Few studies have attempted to determine the mechanism of neutrophil recruitment in cHL. It has been suggested that HRS cells, by secreting lymphotoxin-α, activate endothelial cells leading to upregulating of the adhesion molecules intercellular adhesion molecule-1 (ICAM-1), vascular cell adhesion molecule-1 (VCAM-1), and E-selectin.^6,7,9^ Evidence for this process in vivo in cHL was supported by coimmunostaining of endothelial CD31 and hyaluronan^6^—a glycosaminoglycan induced by proinflammatory cytokines, in addition to E-selectin,^9^ in areas rich in HRS cells. Because HRS cells–derived TNF-α in addition to NETs are stimuli for expression of TF—the clotting initiator,^11,18^ it was of interest to determine TF staining in our cases. Our results showed TF staining in the endothelium of all NS cHL cases (100%), while all other subtypes and reactive conditions were essentially negative. Notably, endothelial TF staining was consistently present in areas rich in HRS cells, variably positive for NETs, but not in areas of the same node (evaluated in the same slide) with rare or absent HRS cells (tumor-poor areas). While we cannot exclude staining is due to TF-containing microparticles,^16,18,19^ it is remarkable that TF gene expression is more frequent in cHL when compared with other lymphoid neoplasms.^42^ In addition, the presence of a procoagulant environment is supported by intra- and extravascular fibrin deposition, endothelial cell activation (VCAM-1, E-selectin, and TF), and platelet thrombi in NS cHL.^4,7,8^ At last, platelets may also contribute to neutrophil recruitment to the node via high-endothelial venules, among other mechanisms.^12,43^ These findings support the view that HRS cells, cytokines, proteases, NETs, and TF potentially promote endothelial cell activation and fuel the coagulation-inflammation cycle generating signals required for lymphocytes and neutrophil recruitment.^2,11,16,28,44^ Conceivably, these events contribute to an inflammatory TME, in a similar manner as conceptually described for immunothrombosis.^28^

NET formation has been implicated in promoting fibrosis by activation of myofibroblasts, which release extracellular matrix proteins that contribute to cardiac fibrosis and heart failure.^21^ These findings are relevant in NS cHL which is often associated with band-like dense fibrosis.^1–3^ In our series, NETs were more frequent in cases in which a fibrotic background was noted as opposed to NET-negative cases, either neoplastic (eg, NLPHL) or reactive. Of note, we found a statistically significant correlation between NET formation and fibrosis. Although a definitive association between these 2 processes remains to be demonstrated, these findings suggest that ETs might contribute to fibrosis observed in the disease, together with transforming growth factor-β (TGF-β), among other mechanisms. Our findings also showed variable degrees of necrosis in about 50% of our cases. At present, it is unclear whether NETs contribute to necrosis and vice-versa, or whether a temporal relationship exists between these 2 mechanisms of cell death.

In conclusion, our data suggest that a hitherto unrecognized inflammatory process mediated by NETs in particular, and immunothrombosis in general, takes place in NS cHL. Additional studies are required to determine whether NETs and the PAR/protease axis represent a viable therapeutic target to decrease tumor growth in cHL.^45,46^ Further studies will be required to extend these observations in a larger cohort of cHL, preferably in the context of clinical trial data to confirm their clinical relevance.

## Acknowledgments

We thank Dr. N.A. Lee (Mayo Clinic, AZ) for EPX antibody. We thank Simon Watkins (Center for Biologic Imaging, University of Pittsburgh) for providing instrumentation (grant 1S10OD019973-01).

## Sources of funding

This research was supported by the Intramural Research Program of the NIH, NCI. This work was partially supported by NIH grant R01 HL123096 (P.I. Ivona Pandrea).

## Disclosures

The authors have no conflicts of interest to disclose.

## Supplementary Material


