# Supplementary material for: Neutrophil and Eosinophil Extracellular Traps in Hodgkin Lymphoma
Source: Hemasphere. 2021 Sep 1;5(9):e633. doi: 10.1097/HS9.0000000000000633 (PMC8410234; doi:10.1097/HS9.0000000000000633)
Supplement: Supplementary file 1 [file hs9-5-e633-s001.pdf]

SUPPLEMENTAL DATA - FIGURE LEGENDS

Neutrophil and Eosinophil Extracellular Traps in Hodgkin Lymphoma

Ivo M.B. Francischetti[1,#], Julie C. Alejo[1,*], Ranjit Sivanandham[2,*], Theresa Davies-Hill[1], Patricia Fetsch[1], Ivona Pandrea[2,3], Elaine S. Jaffe[1] & Stefania Pittaluga[1,#]

Running Title: NETs in Hodgkin Lymphoma

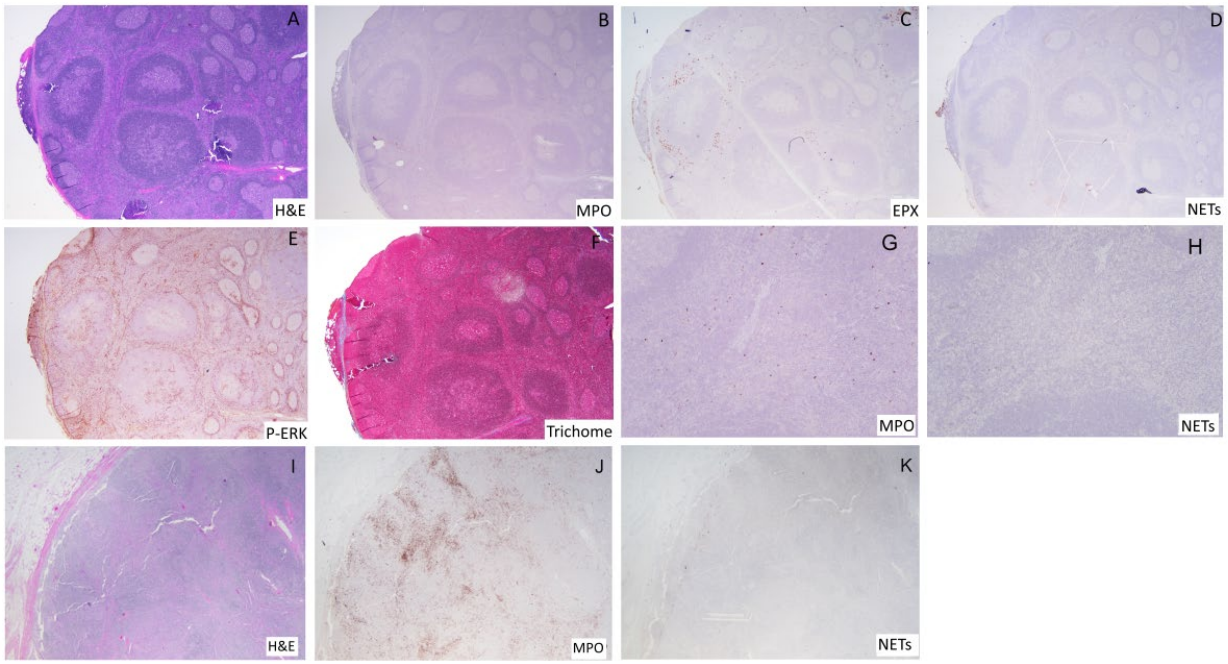

Figure S1

FIGURE S1. NETs are not detectable in Progressive Transformation of Germinal Centers or in Mixed Cellularity cHL.

Case#28 (A-H). (A) H&E, (B) MPO, (C) EPX, (D) Citrullinated histones, (E) *p*-ERK and (F) Trichrome Masson. All figures (x20). (G) MPO, (H) Citrullinated histones (both x100). Mixed cellularity cHL case (I-K). (I) H&E, (J) MPO, (K) Citrullinated histones (all x20). No staining for citrullinated histones is noted in both conditions.

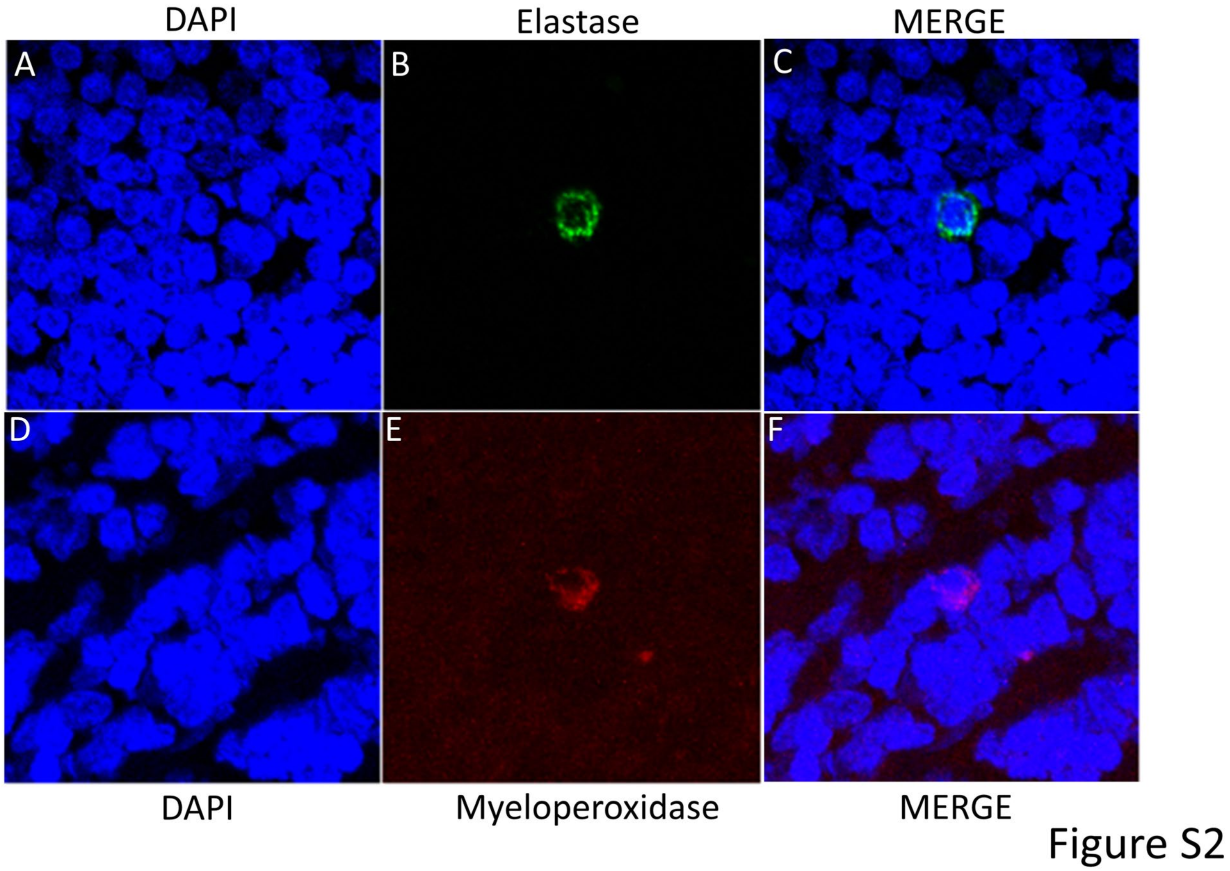

FIGURE S2. NETS are not detectable in NLPHL, by immunofluorescence microscopy. Case #25. (A&D) Nuclear material identification by DAPI (blue), visualization of (B) neutrophil elastase (red) and (E) MPO (green), and Merge shows colocalization of (C) DAPI-stained DNA and elastase and (F) DAPI-stained DNA and MPO. Scale bars are 20 μm in length.

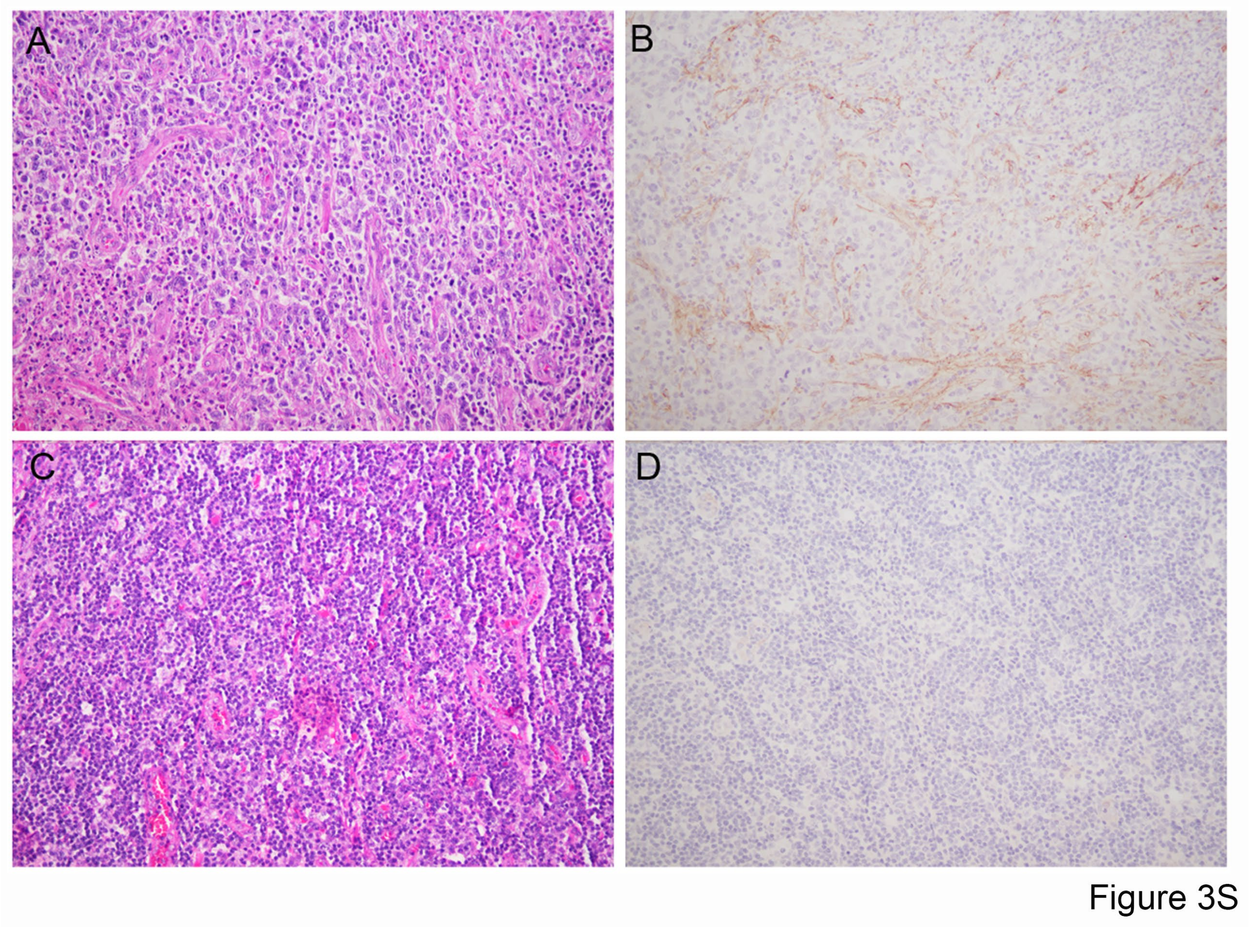

FIGURE S3. TF staining in the endothelium of NS cHL in areas with high HRS cell density. NS cHL case #9. (A) H&E of lymph node with numerous HRS cells in an inflammatory background (high-density tumor areas) (x200). (B) Positive staining for TF in the endothelial cells in high-density tumor density areas (x200). (C) H&E of lymph node with rare HRS cells in a non-inflammatory background (low-density tumor areas)(x200). (D) Negative staining for TF in the endothelial cells in low-density tumor areas (x200).

TABLE 1S: Extended pathology findings and clinical presentation of patients studied.
